# Local Application of Tanshinone IIA protects mesenchymal stem cells from apoptosis and promotes fracture healing in ovariectomized mice

**DOI:** 10.1186/s13018-024-04793-x

**Published:** 2024-05-23

**Authors:** Shao Cheng, Xiaohui Hu, Kanghui Sun, Ziyu Huang, Yongjian Zhao, Yueli Sun, Bo Zeng, Jing Wang, Dongfeng Zhao, Sheng Lu, Qi Shi, Yongjun Wang, Weian Zhang, Xinhua Liu, Bing Shu

**Affiliations:** 1 Longhua Hospital, Shanghai University of Traditional Chinese Medicine, Shanghai, 200032 China; 2 Spine Institute, Shanghai Academy of Traditional Chinese Medicine, Shanghai, 200032 China; 3 Key Laboratory, Ministry of Education of China, Shanghai, 200032 China; 4 School of Orthopedics, Henan University of Chinese Medicine, Zhengzhou, 450002 China; 5 Shanghai Key Laboratory of Functional Materials Chemistry, East China University of Science and Technology, Shanghai, 200237 China; 6 Shanghai University of Traditional Chinese Medicine, Shanghai, 201203 China

**Keywords:** Tanshinone IIA, Osteoporotic fracture, Injectable hydrogel, Mesenchymal stem cell, Oxidative stress, Cell apoptosis, Nuclear factor erythroid 2-related factor 2

## Abstract

**Background:**

Elderly patients suffering from osteoporotic fractures are more susceptible to delayed union or nonunion, and their bodies then are in a state of low-grade chronic inflammation with decreased antioxidant capacity. Tanshinone IIA is widely used in treating cardiovascular and cerebrovascular diseases in China and has anti-inflammatory and antioxidant effects. We aimed to observe the antioxidant effects of Tanshinone IIA on mesenchymal stem cells (MSCs), which play important roles in bone repair, and the effects of local application of Tanshinone IIA using an injectable biodegradable hydrogel on osteoporotic fracture healing.

**Methods:**

MSCs were pretreated with or without different concentrations of Tanshinone IIA followed by H_2_O_2_ treatment. Ovariectomized (OVX) C57BL/6 mice received a mid-shaft transverse osteotomy fracture on the left tibia, and Tanshinone IIA was applied to the fracture site using an injectable hydrogel.

**Results:**

Tanshinone IIA pretreatment promoted the expression of nuclear factor erythroid 2-related factor 2 and antioxidant enzymes, and inhibited H_2_O_2_-induced reactive oxygen species accumulation in MSCs. Furthermore, Tanshinone IIA reversed H_2_O_2_-induced apoptosis and decrease in osteogenic differentiation in MSCs. After 4 weeks of treatment with Tanshinone IIA in OVX mice, the bone mineral density of the callus was significantly increased and the biomechanical properties of the healed tibias were improved. Cell apoptosis was decreased and Nrf2 expression was increased in the early stage of callus formation.

**Conclusions:**

Taken together, these results indicate that Tanshinone IIA can activate antioxidant enzymes to protect MSCs from H_2_O_2_-induced cell apoptosis and osteogenic differentiation inhibition. Local application of Tanshinone IIA accelerates fracture healing in ovariectomized mice.

## Background

Patients suffering from osteoporotic fractures, especially elderly individuals, are more susceptible to delayed union or nonunion, resulting in weakened biomechanical properties. Osteoporotic fractures represent the most serious complication of osteoporosis and are associated with higher disability and mortality rates than general fractures [1]. It is widely thought that by 2050, half of the global osteoporotic hip fractures will occur in Asia, imposing an enormous economic burden on medical and health expenditures [2].

During the early stages of fracture healing, the local environment, characterized by ischemia, hypoxia, and inflammation, results in the accumulation of reactive oxygen species (ROS), leading to increased oxidative stress levels. Generally, the antioxidant system clears these ROS to protect cells from oxidative damage [3–5]; however, in patients with osteoporosis, due to ageing, estrogen levels decline, and other factors, the body is in a state of low-grade chronic inflammation, and the antioxidant capacity of the body is decreased [6, 7]. Consequently, excessive and prolonged ROS activity at the fracture site can lead to cell cycle arrest and decreased osteogenic differentiation [8, 9], which are critical for bone regeneration during fracture healing.

Tanshinone IIA (CAS No. 568-72-9) is widely used for treating cardiovascular and cerebrovascular diseases in China. Tanshinone IIA has been confirmed to have anti-inflammatory, antioxidant, and other pharmacological effects [10–12]. Further studies with nerve cells, acinar cells, lung tissue, and myocardium revealed the involvement of the Kelch-like ech-associated protein-1 (Keap1)-nuclear factor erythroid 2-related factor 2 (Nrf2) pathway in the protective effects of Tanshinone IIA against ROS [13–15]. In addition, Tanshinone IIA can increase the recruitment of bone marrow mesenchymal stem cells (MSCs) and promote the osteogenic differentiation of MC3T3-E1 cells [16, 17]. Based on these findings, we hypothesized that Tanshinone IIA might be a potential candidate for treating osteoporotic fractures. Most of the previous studies on bone repair have focused on defects in flat bones or cortical bones, while there have been few studies on the treatment of long bone osteoporotic fractures, which have greater potential for clinical application.

In this study, we applied Tanshinone IIA to the fracture site in ovariectomized (OVX) mice using an injectable hydrogel and observed the effects of the local application of Tanshinone IIA on osteoporotic fracture healing. We also performed in vitro experiments to confirm the antioxidant effects of Tanshinone IIA on bone marrow MSCs.

## Methods

### Cell survival rate assessment

Tanshinone IIA (CAS No. 568-72-9) was purchased from Yuanye Biotechnology Co., Ltd (Shanghai, China). The purity of Tanshinone IIA was confirmed to be more than 98% by high-performance liquid chromatography. Primary bone marrow MSCs were plated in a 96-well plate at a density of 5 × 10^3^ cells/well for 24 h and further treated with 0, 10^− 9^, 10^− 8^, 10^− 7^, 10^− 6^ or 2 × 10^− 6^M Tanshinone IIA/dimethyl sulfoxide (DMSO) solutions for another 24 h. For H_2_O_2_ treatment, cells were plated in a 96-well plate at a density of 2 × 10^3^ cells/well for 24 h and were pretreated with 2 × 10^− 6^M Tanshinone IIA or DMSO for another 24 h. Then, the medium was discarded, and the cells were incubated with 0, 50, 100, 200, 400, 500, 600, 800, or 1000µM H_2_O_2_ for 24 h. The cell survival rate was assessed with an enhanced cell counting kit-8 (C0041, Beyotime Biotechnology, Shanghai, China), and the absorbance at 450 nm was recorded by a microplate reader.

### Osteogenic differentiation assay

Bone marrow cells from long bones of 4-week-old C57BL/6 mice were collected and plated in αMEM supplemented with 10% fetal bovine serum and 1% penicillin–streptomycin. After 7 days of culture, the suspended cells were removed, and the adherent cells were collected and plated in a 12-well plate at a density of 8 × 10^4^ cells/well with osteogenic differentiation medium containing 50 mg/L vitamin C, 10nM dexamethasone, and 10mM β-glycerophosphate in complete αMEM. The cells were incubated with different concentrations of Tanshinone IIA with or without 500µM H_2_O_2_. The culture medium was refreshed every 3 days. After 10 days or 21 days culture, the cells were fixed with 10% buffered formalin for 15 min and stained with 1-step NBT/BCIP substrate solution (34,042, Thermo, Waltham, Massachusetts, USA) for 30 min or 0.1% Alizarin red (A5533, Sigma, St. Louis, Missouri, USA) aqueous solution for 30 min.

### Real-time reverse transcriptase polymerase chain reaction (RT–PCR) assay

Primary bone marrow MSCs were plated in a 6-well plate at a density of 2 × 10^5^ cells/well with osteogenic differentiation medium. Cells were treated with either 500µM H_2_O_2_ or 500µM H_2_O_2_ and different concentrations of Tanshinone IIA for 72 h. Total RNA of the cells was extracted using an RNA Extraction Kit (B0004D, HifunBio, Shanghai, China) and reverse transcribed to cDNA using an RT reagent kit (RR407, Takara Bio, Japan). PCR was performed using a TB Green Premix Ex Taq II kit (RR820, Takara Bio, Japan). The primers used for specific mRNAs are listed in Table 1.

**Table 1:** PCR primers for specific genes

Genes	primer sequences		
*β-actin*	Forward	5’- TATCGCTGCGCTGGTCG − 3’	
	Reverse	5’- CCCACGATGGAGGGGAATAC − 3’	
*Runx2*	Forward	5’- GTGGCAGTGTCATCATCTGAAAT − 3’	
	Reverse	5’-TCGCCTCAGTGATTTAGGGCGCA-3’	
*osterix*	Forward	5’- TGCTATACTCTGGGGGCTCTC − 3’	
	Reverse	5’- AGGAGGTCGGAGCATAGGAA − 3’	

### Western blotting assay

C_3_H_10_T_1/2_ cells provided by the National Collection of Authenticated Cultures, Chinese Academy of Sciences were plated in a 6-well plate at a density of 8 × 10^4^ cells/well for 24 h and were pretreated with 0, 10^− 8^, 10^− 7^, or 10^− 6^M Tanshinone IIA for 24 h. After incubation in 500µM H_2_O_2_ for another 24 h, the cells were collected, and total proteins were extracted on ice using radioimmunoprecipitation assay lysis buffer (P0013B, Beyotime Biotechnology, Shanghai, China). The protein concentration was detected using an enhanced bicinchoninic acid assay kit (P0010, Beyotime Biotechnology, Shanghai, China). Western blot analysis was conducted as previously described [18]. The following antibodies were used: anti-cleaved caspase 3 (ab214430), anti-pro caspase 3 (ab32499), anti-superoxide dismutase 1 (SOD 1, ab13498), anti-heme oxygenase-1 (HO-1, ab68477), anti-catalase (CAT, ab16731) from Abcam (Cambridge, UK) and anti-Nrf2 (12721), anti-β-actin (8457), anti-Keap1 (8047), anti-Bcl2 (3498) and anti-Bax (5023) from Cell Signaling Technology (Danvers, Massachusetts, USA). A chemiluminescent horseradish peroxidase substrate kit (WBKLS0500, Millipore, Billerica, Massachusetts, USA) was used for the electrochemiluminescence detection assay.

### Detection of antioxidant enzymes

C_3_H_10_T_1/2_ cells were plated in a 12-well plate or 6-well plate for 24 h. After pretreatment with different concentrations of Tanshinone IIA or DMSO for 24 h, the cells were incubated in fresh medium supplemented with 500µM H_2_O_2_ for another 24 h. For ROS detection, the cells were incubated in αMEM containing a 10mM 2,7-dichlorodihydrofluorescein diacetate (H_2_DCFDA) fluorescence probe (287810, Sigma, St. Louis, Missouri, USA) for 30 min, and the fluorescence intensity was measured by flow cytometry (Accuri C6, BD Biosciences, Franklin Lakes, New Jersey, USA). SOD and CAT activity in cells was detected using relative activity test kits (SOD, S0101S; CAT, S0051; Beyotime Biotechnology, Shanghai, China).

### Immunofluorescence staining

C_3_H_10_T_1/2_ cells were plated in a 48-well plate and cultured with 0, 10^− 8^, 10^− 7^, or 10^− 6^M Tanshinone IIA for 24 h, followed by culture in fresh medium supplemented with 500µM H_2_O_2_ for another 24 h. After fixation with 4% formaldehyde solution at 37℃ for 15 min, the cells were incubated with an Nrf2 antibody (12721, Cell Signaling Technology, Danvers, Massachusetts, USA, 1:500) overnight at 4℃. Cells were further incubated in CoraLite594-conjugated goat anti-rabbit IgG (SA00013-4; Proteintech, Wuhan, Hubei, China; 1:200) for 1 h at 37℃ in the dark. Antifade mounting medium with 4,6-diamidino-2-phenylindole (DAPI) was used for nuclear fluorescence staining (H-1200, Vector Laboratories, San Francisco, California, USA).

### Animals

The study was conducted according to the guidelines of the Declaration of Helsinki, and approved by the Institutional Animal Care and Use Committee of Longhua Hospital, Shanghai University of Traditional Chinese Medicine (2019-N051). Three-month-old female C57BL/6 mice (21–23 g body weight) ordered from Lingchang Biotech (Shanghai, China) were housed at 22˚C with a 12 h light/dark cycle, with 4 or fewer mice in each cage, and were provided *ad libitum* access to food and water in the specific pathogen-free animal experiment center of Longhua Hospital.

### Grouping and model establishment

Following a 1-week acclimatization phase, the mice were randomly assigned to the sham, model, gel, and Tanshinone IIA groups using a random number table. Mice in the model, gel, and Tanshinone IIA groups underwent bilateral ovariectomy, and the rest underwent sham operations. Three months after the surgery, all mice received a mid-shaft transverse osteotomy fracture on the left tibias, and the tibias were fixed with 0.5-mm-diameter intramedullary metallic pins. Anesthesia was induced in mice by isoflurane inhalation through an inhalation anesthesia machine before surgery.

### Hydrogel preparation and intervention

The injectable hydrogel was prepared as previously described [19]. A filtered 40 mg/ml dextran/phosphate buffered saline (PBS) solution containing 4µM Tanshinone IIA/DMSO or an equal volume of DMSO and 20 mg/ml filtered chitosan/PBS solution was prepared. When setting up the fracture model, the dextran/PBS solution and chitosan/PBS solution were mixed at a 1:1 ratio, and 20 µl of semisolidified hydrogel was injected into the bilateral bone marrow cavities of the fracture ends before fixing the tibias with pins. Mice in the Tanshinone IIA group were injected with a hydrogel containing 2µM Tanshinone IIA, and mice in the gel group received a hydrogel containing DMSO.

### Sample harvest

Mice were sacrificed at the corresponding time points. Mice were anesthetized with isoflurane in an induction chamber, followed by intraperitoneal injection of pentobarbital sodium (5.4 g/kg body weight). The left tibias (6 from each group per time point) were fixed in 10% buffered formalin and subjected to micro-computed tomography (micro-CT) scanning. After decalcification, dehydration, and embedding, 4 μm thick serial sections of the tibias were cut for histomorphometric evaluation. Another set of 5 tibias from each group was harvested 28 days postfracture and stored at -80°C for biomechanical testing.

### Biomechanical test

The tibias were equilibrated with saline at room temperature before assessment. A three-point mechanical bending test was performed on the left tibias to assess the maximum force and yield displacement with a mechanical testing instrument (ElectroForce 3200 Series III, TA Instrument, New Castle, Delaware, USA).

### Threedimensional (3D) reconstruction analyses

The fractured tibias were subjected to X-ray imaging and micro-CT scanning (vivaCT 40, Scanco Medical AG, Brüttisellen, Switzerland) successively at a voltage of 55 kV and a current of 72µA. The integration time was 300ms, and the slice increment was 10 mm. The bone volume (BV, mm^3^), total volume (TV, mm^3^), bone mineral density (BMD), trabecular bone number (Tb.N), trabecular bone thickness (Tb.Th), and trabecular bone separation (Tb.Sp) of the callus were measured.

### Histological evaluation

For morphometric analysis, midsagittal sections of the tibias were dewaxed and stained with Alcian blue/hematoxylin solution and orange G solution. After dehydration, clearing, and mounting, images of the sections were captured by a virtual slide system (VS120-S6-W, Olympus, Japan).

### TdT-mediated dUTP Nick End Labelling (TUNEL)

C_3_H_10_T_1/2_ cells were plated in a 48-well plate at a density of 1 × 10^4^ cells/well for 24 h and were pretreated with 0, 10^− 8^, 10^− 7^, or 10^− 6^M Tanshinone IIA for 24 h. After incubation in 500µM H_2_O_2_ for another 24 h, cells were fixed with 4% formaldehyde at 37℃ for 30 min. For the animal study, midsagittal sections of the callus were dewaxed and rehydrated. TUNEL staining was performed using a staining kit (C1088, Beyotime Biotechnology, Shanghai, China), and cell apoptosis was observed using a fluorescence microscope and were analyzed with an image Pro Plus 6.0 software (Media Cybernetics, PA, USA).

### Immunohistochemical staining

After dewaxing and rehydration, sections were treated with 3% H_2_O_2_ solution for 15 min at 37 °C, followed by the antigen repair with sodium citrate solution for 15 min at 95 °C. The sections were incubated in primary Nrf2 antibody (16396-1-AP, Wuhan Sanying, Wuhan, China, 1:100) overnight at 4 °C. The sections were then incubated in horseradish peroxidase labeled goat anti rabbit IgG (A0208, Beyotime Biotechnology, Shanghai, China) for 40 min. After staining with diaminobenzidine and counterstaining with hematoxylin, the images were captured by by a virtual slide system (VS120-S6-W, Olympus, Japan), and were analyzed with an image Pro Plus 6.0 software (Media Cybernetics, PA, USA).

### Statistical analyses

All of the data are presented as the means ± standard deviations. Statistical analyses and graph generation were performed using GraphPad Prism 8 (GraphPad Software, San Diego, California, USA). The normality of the data was assessed by the Shapiro‒Wilk test, and the homogeneity of variance was tested. For data with homogeneity and normality, statistical significance among multiple groups was assessed by one-way analysis of variance (ANOVA). Otherwise, the Kruskal‒Wallis H test was used. A P value < 0.05 was considered to indicate statistical significance.

## Results

### Tanshinone IIA mitigated H_2_O_2_-induced apoptosis in MSCs

**Fig. 1:**
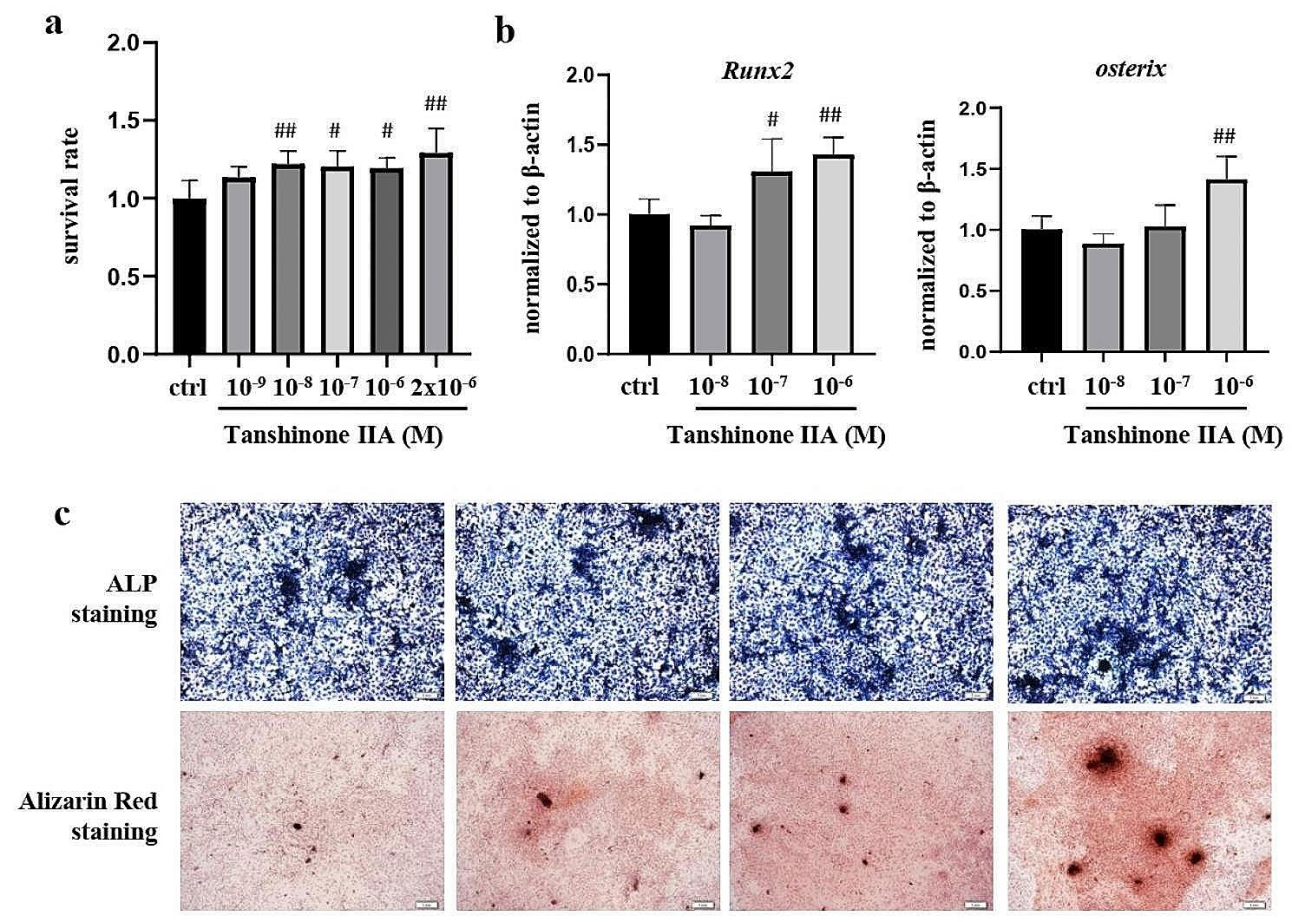
Effects of Tanshinone IIA on the osteogenic differentiation of primary bone marrow MSCs. Primary bone marrow MSCs were treated with different concentrations of Tanshinone IIA for 24 h. **(a)** Survival rates of primary bone marrow MSCs. **(b)** The gene expression levels of Runx2 and osterix in primary bone marrow MSCs. **(c)** Images of ALP staining and Alizarin Red staining of primary bone marrow MSCs. MSC, mesenchymal stem cell. ALP, alkaline phosphatase. All experiments were repeated at least 3 times. Compared with the ctrl group, #, *p* < 0.05; ##, *p* < 0.01

To assess the safety of Tanshinone IIA, primary bone marrow MSCs were treated with different concentrations of Tanshinone IIA solutions. Tanshinone IIA solution at a concentration of 2 × 10^-6^M or less showed no cytotoxicity compared with the negative control group (Fig. 1a). When bone marrow MSCs were treated with Tanshinone IIA, *Runx2* (10^-6^M and 10^-7^M groups) and *osterix* (10^-6^M groups) expression levels were slightly increased (Fig. 1b). Additionally, the osteogenic differentiation ability and mineralization ability of the cells were slightly increased in the group treated with the high concentration of Tanshinone IIA (Fig. 1c).

**Fig. 2:**
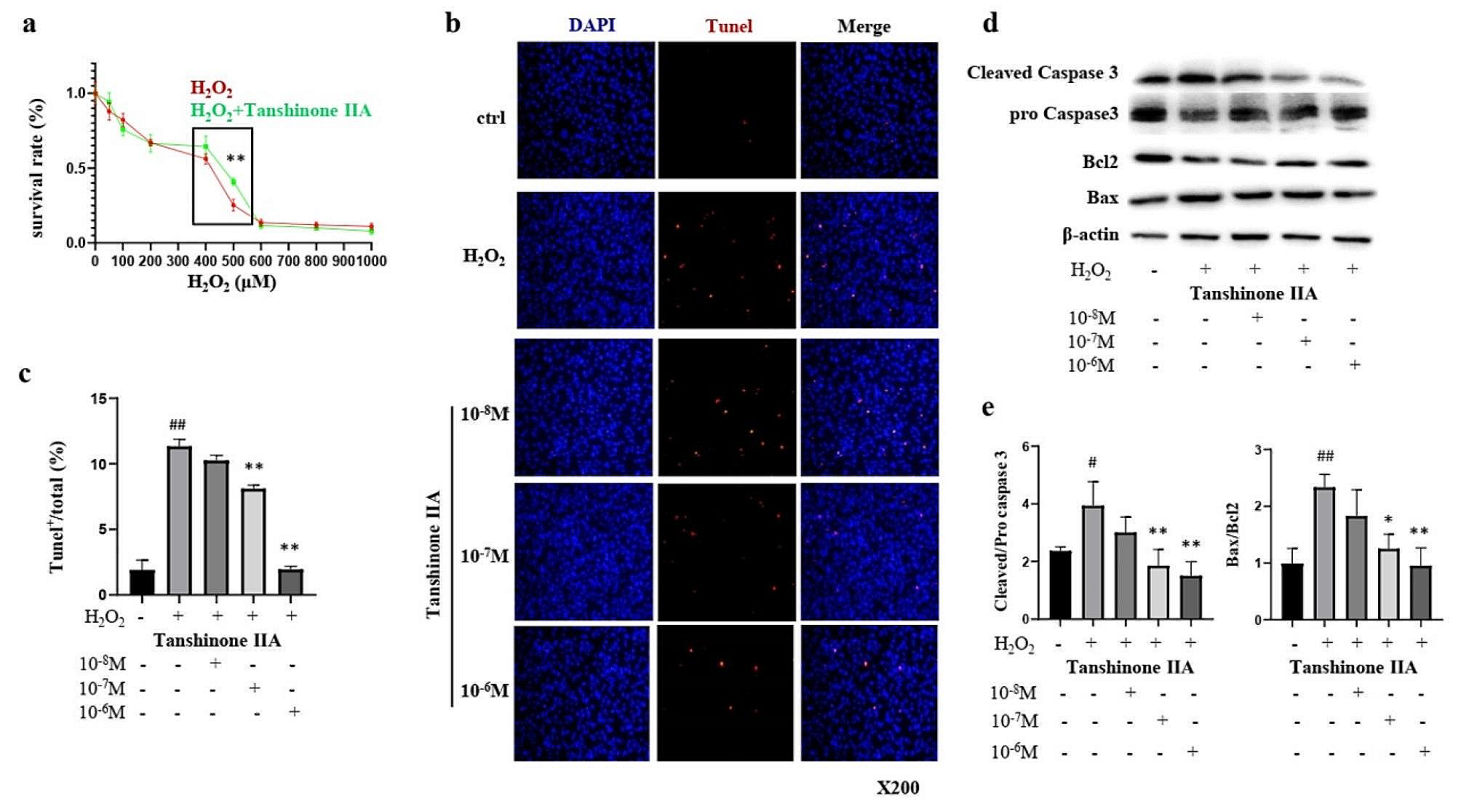
Tanshinone IIA inhibited H_2_O_2_-induced apoptosis in MSCs. **a)** Survival rates of C_3_H_10_T_1/2_ cells sequentially pretreated with 2 × 10^-6^M Tanshinone IIA for 24 h and different concentrations of H_2_O_2_ for 24 h. **b**, **c**) C_3_H_10_T_1/2_ cells were pretreated with different concentrations of Tanshinone IIA for 24 h and then treated with 500µM H_2_O_2_ for another 24 h. TUNEL staining for cell apoptosis and quantification of the proportions of TUNEL^+^ cells among the total cells are shown. **d**, **e**) C_3_H_10_T_1/2_ cells were pretreated with different concentrations of Tanshinone IIA for 24 h and then treated with 500 µM H_2_O_2_ for another 24 h. Western blotting assays and quantification for cell apoptosis-related proteins are shown. MSC, mesenchymal stem cell. TUNEL, TdT-mediated dUTP nick end labelling method. All experiments were repeated at least 3 times. H_2_O_2_ group vs. negative control group, #, *p* < 0.05; ##, *p* < 0.01. Tanshinone IIA groups vs. H_2_O_2_ group, *, *p* < 0.05; **, *p* < 0.01

After treatment with different concentrations of H_2_O_2_ for 24 h, the percentage of surviving cells gradually decreased with increasing H_2_O_2_ concentration, with the most significant decrease observed in the range of 300µM–600µM H_2_O_2_. However, pretreatment with 2 × 10^-6^M Tanshinone IIA for 24 h significantly reversed the decrease in survival rate in 500µM H_2_O_2_-treated cells, suggesting that Tanshinone IIA has a protective effect on cell survival within a certain range (Fig. 2a). TUNEL staining revealed that 500µM H_2_O_2_ induced significant cell apoptosis, while pretreatment with a high dose of Tanshinone IIA (10^-7^M and 10^-6^M) for 24 h significantly decreased H_2_O_2_-induced apoptosis in C_3_H_10_T_1/2_ cells (Fig. 2b and c). Changes in the expression levels of cell apoptosis-related proteins were further investigated. Pretreatment with a high dose of Tanshinone IIA inhibited the H_2_O_2_-induced increase in cleaved caspase 3/pro caspase 3 and Bax/Bcl2 in C_3_H_10_T_1/2_ cells (Fig. 2d and e).

### Tanshinone IIA reversed the H_2_O_2_-induced decrease in osteogenic differentiation in primary bone marrow MSCs

**Fig. 3:**
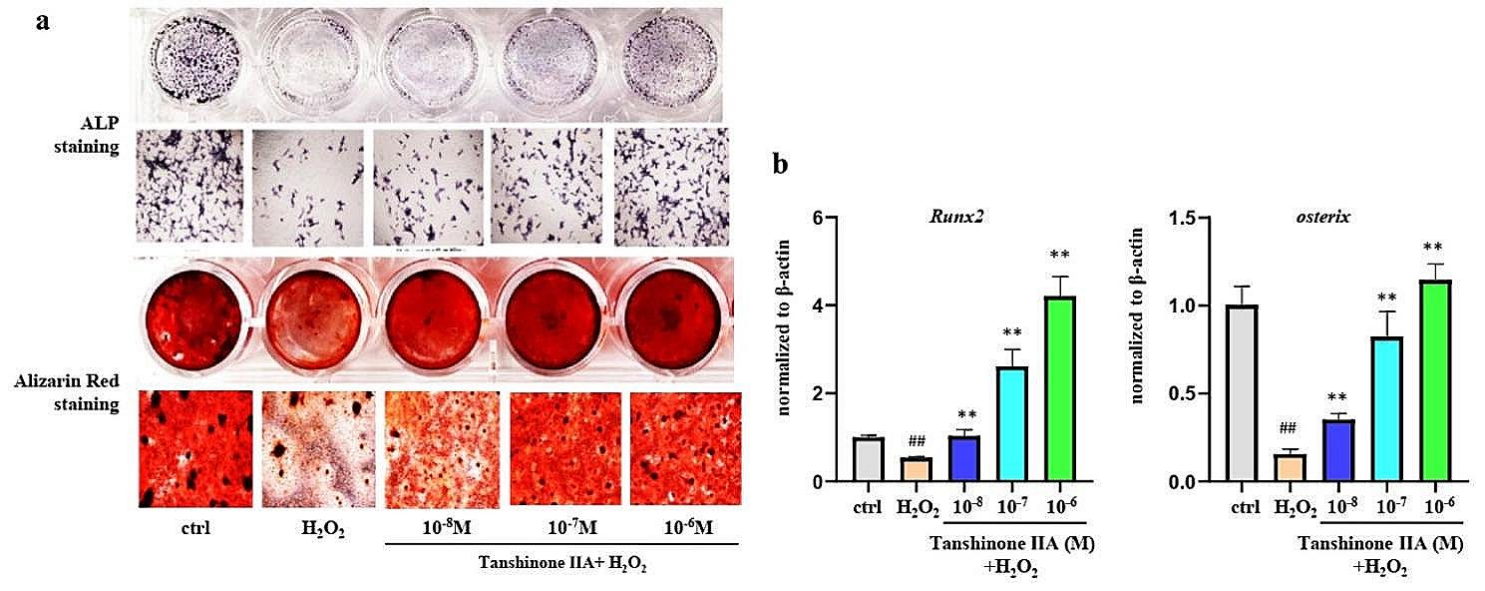
Tanshinone IIA reversed the H_2_O_2_-induced decrease in osteogenic differentiation in primary bone marrow MSCs. Primary bone marrow MSCs pretreated with Tanshinone IIA for 24 h were further treated with 500µM H_2_O_2_. **(a)** Images of ALP staining and Alizarin Red staining showing the osteogenic differentiation and mineralization of primary bone marrow MSCs. **(b)** The gene expression levels of Runx2 and osterix in primary bone marrow MSCs. MSC, mesenchymal stem cell. ALP, alkaline phosphatase. All experiments were repeated at least 3 times. H_2_O_2_ group vs. ctrl group, ##, *p* < 0.01. Tanshinone IIA groups vs. H_2_O_2_ group, **, *p* < 0.01

To evaluate the effects of Tanshinone IIA under oxidative stress, primary bone marrow MSCs were further treated sequentially with different concentrations of Tanshinone IIA for 24 h and 500µM H_2_O_2_. H_2_O_2_ treatment dramatically impaired the ability of primary bone marrow MSCs to undergo osteogenic differentiation and mineralization, and pretreatment with Tanshinone IIA reversed the decrease induced by H_2_O_2_ in a dose-dependent manner (Fig. 3a). In addition, the H_2_O_2_-induced decreases in the gene expression levels of Runx2 and osterix were reversed by Tanshinone IIA pretreatment (Fig. 3b).

### Tanshinone IIA promoted Nrf2 and antioxidant enzyme expression in MSCs

**Fig. 4:**
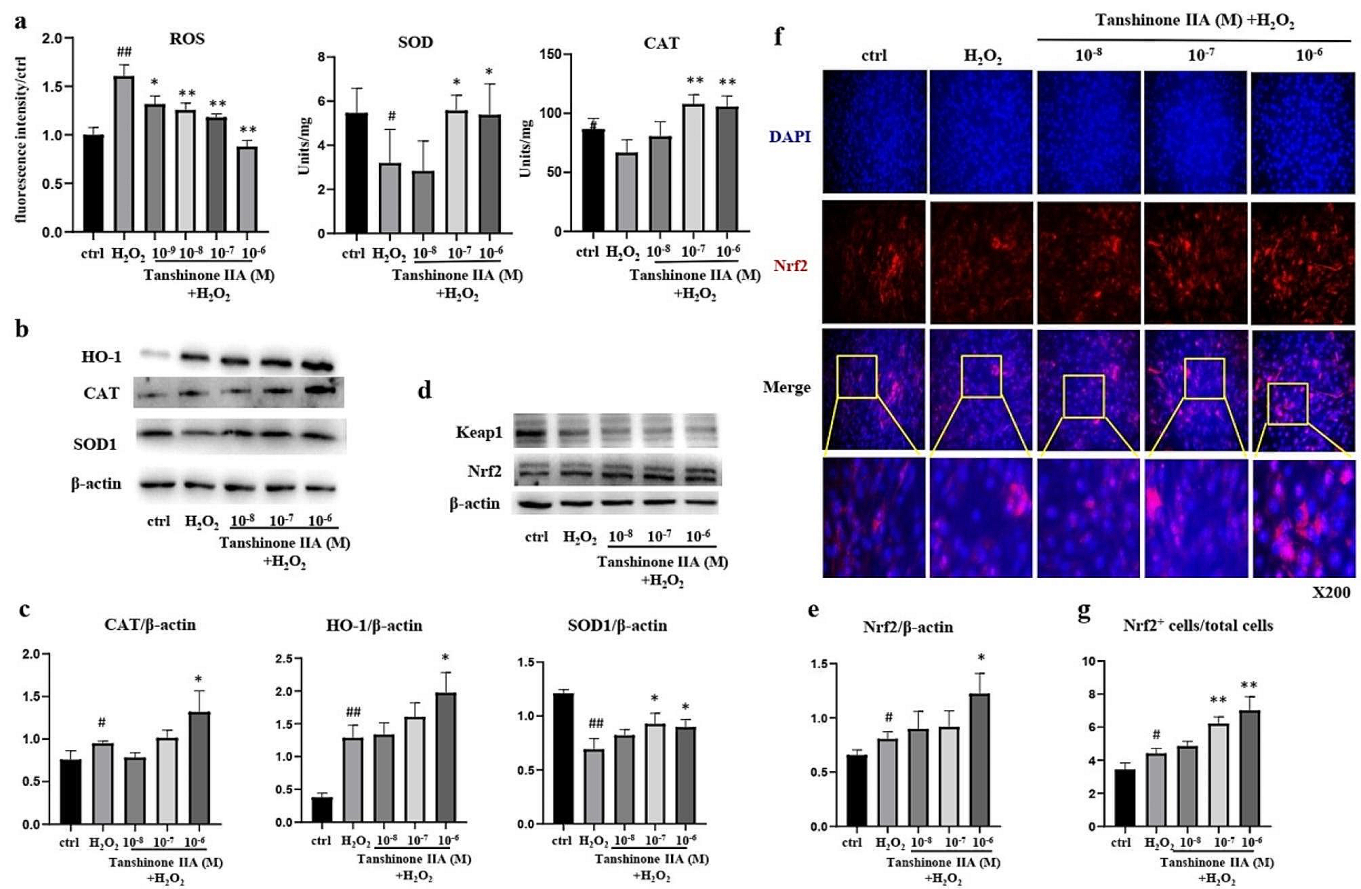
Tanshinone IIA increased Nrf2 and antioxidant enzyme expression in C_3_H_10_T_1/2_ cells. C_3_H_10_T_1/2_ cells were pretreated with different concentrations of Tanshinone IIA for 24 h, followed by stimulation with 500µM H_2_O_2_ for another 24 h. **a)** ROS levels and the enzyme activities of SOD and CAT in C_3_H_10_T_1/2_ cells. **b**, **c**) Western blotting assays and quantification of SOD, HO-1, and CAT in C_3_H_10_T_1/2_ cells. **d**, **e**) Western blotting assays and quantification of Keap1 and Nrf2 expression in C_3_H_10_T_1/2_ cells. **f**, **g**) Immunofluorescence staining for Nrf2, and quantification of the proportions of Nrf2^+^ cells in total C_3_H_10_T_1/2_ cells. SOD, superoxide dismutase; HO-1, heme oxygenase-1; CAT, catalase; Nrf2, nuclear factor erythroid 2-related factor 2. All experiments were repeated at least 3 times. H_2_O_2_ group vs. ctrl group, #, *p* < 0.05; ##, *p* < 0.01. Tanshinone IIA groups vs. H_2_O_2_ group, *, *p* < 0.05; **, *p* < 0.01

To observe the antioxidant effects of Tanshinone IIA, C_3_H_10_T_1/2_ cells were pretreated with different concentrations of Tanshinone IIA for 24 h, followed by H_2_O_2_ stimulation for another 24 h. Tanshinone IIA significantly inhibited H_2_O_2_-induced ROS accumulation in C_3_H_10_T_1/2_ cells in a dose-dependent manner (Fig. 4a). The activity of antioxidant enzymes in C_3_H_10_T_1/2_ cells, including SOD and CAT, was increased by treatment with a high concentration (10^-6^M) of Tanshinone IIA compared with that in the H_2_O_2_ group (Fig. 4a). Western Blotting assays and quantitative analyses also confirmed increased SOD 1, HO-1, and CAT expression levels in C_3_H_10_T_1/2_ cells (Fig. 4b-c). The Keap1/Nrf2 signalling pathway is the main defence mechanism against oxidative stress mediated by the regulation of antioxidant enzyme synthesis. Western blotting assays demonstrated that treatment with a high concentration of Tanshinone IIA increased Nrf2 expression in H_2_O_2_-treated C_3_H_10_T_1/2_ cells but had no effect on Keap1 expression (Fig. 4d and e). Immunofluorescence staining for Nrf2 also revealed increased Nrf2 expression in C_3_H_10_T_1/2_ cells treated with a high concentration of Tanshinone IIA (Fig. 4f and g).

### Local application of Tanshinone IIA promoted fracture healing in OVX mice

**Fig. 5:**
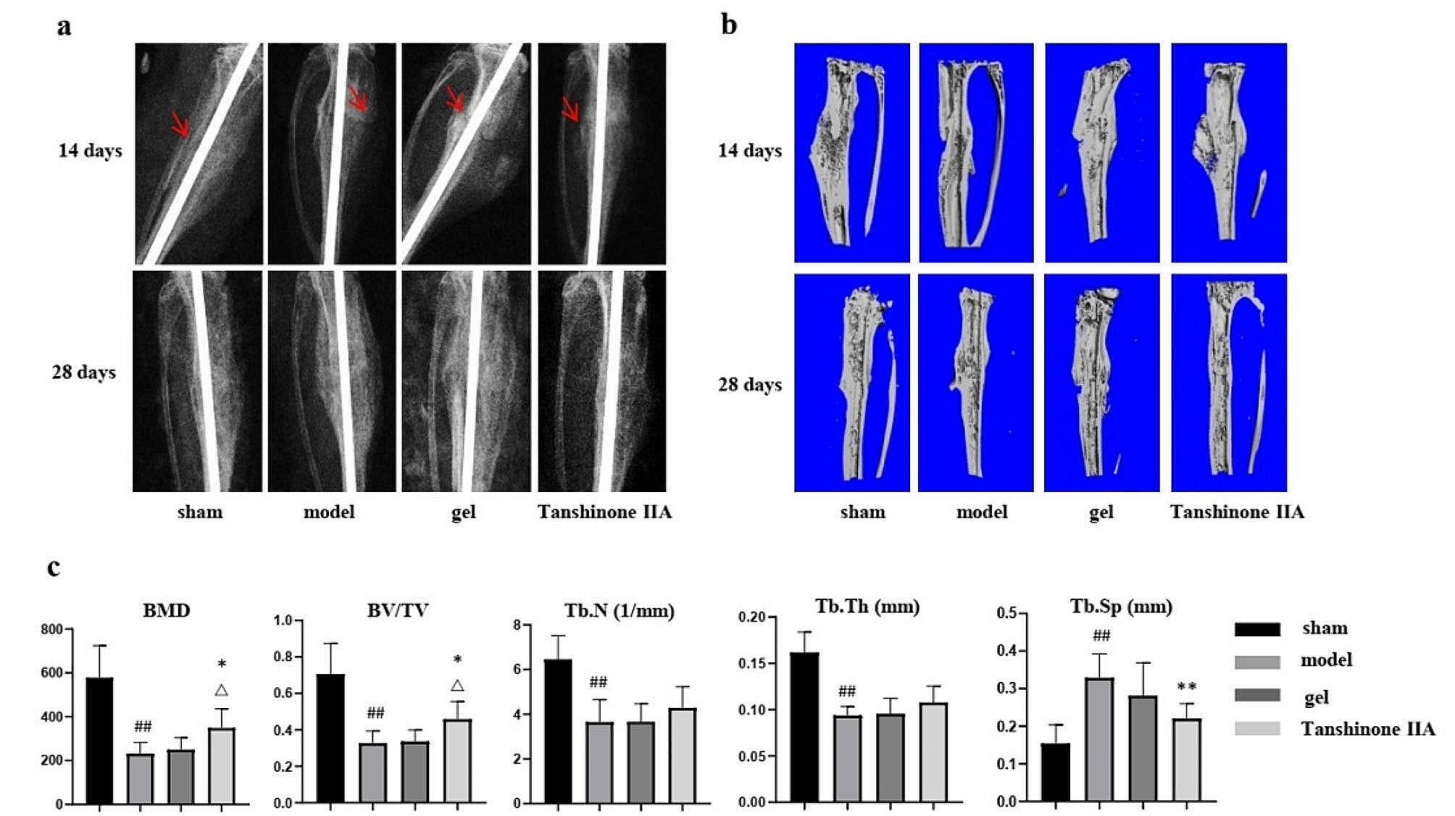
Tanshinone IIA improved the bone mineral density and microstructure of the callus in OVX mice. **(a)** X-ray images of the fracture sites in mice in different groups 14 days and 28 days postfracture. Arrows, fracture lines. **(b)** Sectional views of the callus reconstructed by micro-CT analyses 14 days and 28 days postfracture. **(c)** Quantitative analyses of bone mass and trabecular parameters of the callus in mice in different groups 28 days postfracture. *n* = 6. OVX, ovariectomized. Model group vs. sham group, ##, *p* < 0.01. Tanshinone IIA group vs. model group, *, *p* < 0.05; **, *p* < 0.01. Tanshinone IIA group vs. gel group, △, *p* < 0.05

**Fig. 6:**
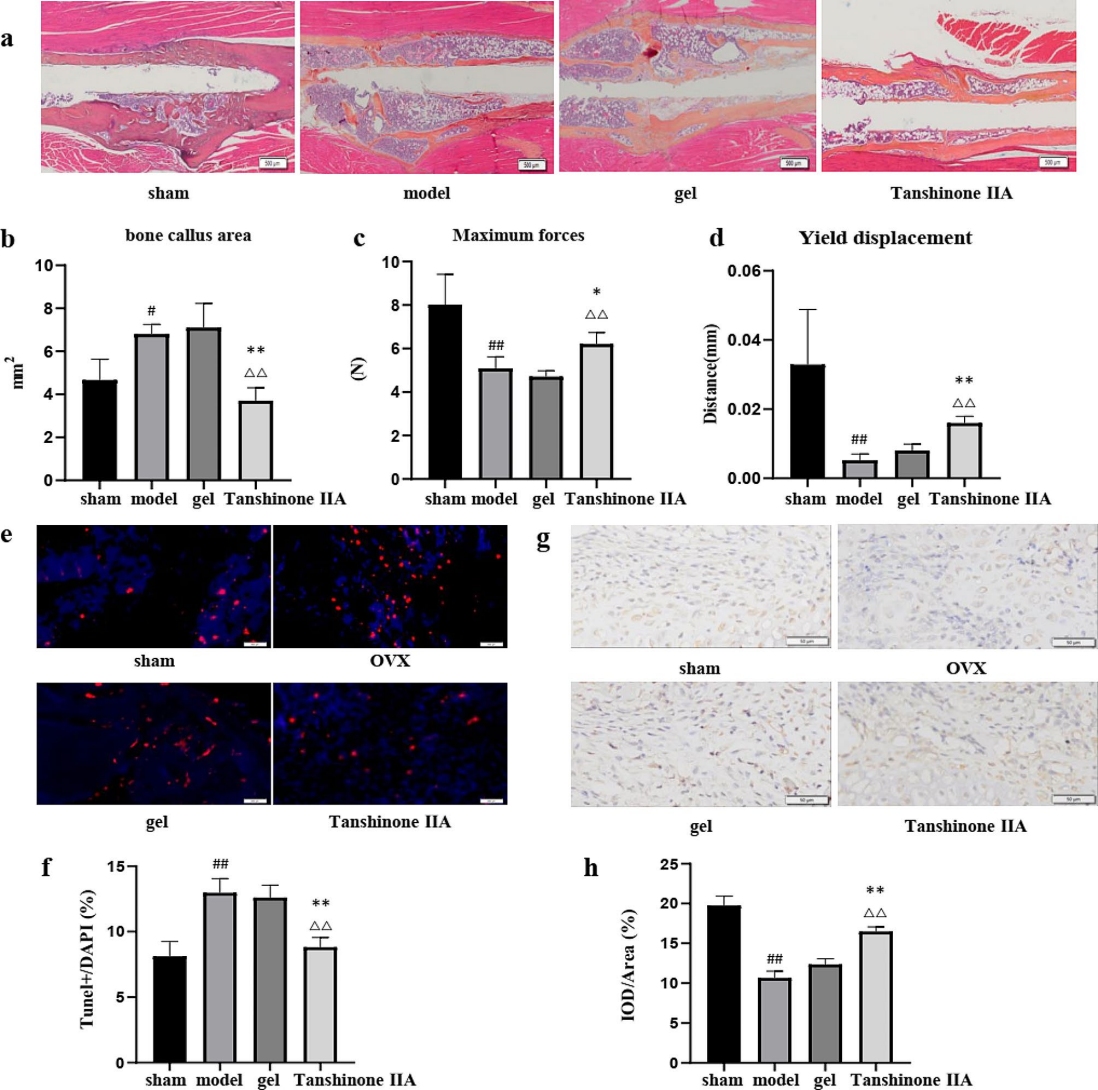
Tanshinone IIA improved the histopathological structure and biomechanical properties of the callus in OVX mice. **a**, **b**) Alcian blue/hematoxylin/orange G staining of the callus and quantitative analyses of the callus area in mice in different groups 28 days postfracture. *n* = 3. Maximum forces **c**) and yield displacement **d**) of the tibias of mice in different groups 28 days postfracture. *n* = 5. **e**, **f**) TUNEL staining of the callus of different groups 7 days postfracture. *n* = 3. **g**, **h**) IHC for Nrf2 expression with the callus of different groups 7 days postfracture. *n* = 3. OVX, ovariectomized. Model group vs. sham group, #, *p* < 0.05; ##, *p* < 0.01. Tanshinone IIA group vs. model group, *, *p* < 0.05; **, *p* < 0.01. Tanshinone IIA group vs. gel group, △△, *p* < 0.01

As shown by the X-ray and 3D reconstruction images, 2 weeks after fracture, significant callus formation was observed in the tibias of mice from the sham group and Tanshinone IIA group, while mice in the model group and gel group exhibited less callus formation. Four weeks after fracture, small calluses with smooth and continuous cortical bone were found in sham mice and Tanshinone IIA-treated mice, while the model and gel groups exhibited large calluses, suggesting accelerated bone fracture healing in OVX mice after Tanshinone IIA treatment (Fig. 5a and b). Compared with those in the sham group, the BMD, BV/TV, Tb.N, and Tb.Th of the callus in the model group were significantly lower, while the Tb. Sp was higher at 4 weeks after the fracture. After 4 weeks of treatment with Tanshinone IIA, the BMD and BV/TV significantly increased compared with those of the model group and gel group (Fig. 5c). Histological staining also confirmed the improved microstructure of the callus with a smaller callus and smoother cortical bone at 28 days postfracture after Tanshinone IIA treatment (Fig. 6a and b). Biomechanical tests revealed greater maximum force and yield displacement in the Tanshinone IIA-treated mice than those in the model mice 4 weeks after treatment (Fig. 6c and d). To confirm the antiapoptotic effect of Tanshinone IIA in vivo, TUNEL staining was performed on 7-day callus from each group. The dramatical increase in cell apoptosis in the callus of the model group was partially alleviated in Tanshinone IIA group (Fig. 6e and f). IHC staining also confirmed the evalutated Nrf2 expression in the callus of the Tanshinone IIA group compared with that of the model group in the early stage of fracture healing (Fig. 6g and h).

## Discussion

Osteoporosis is characterized by an increased level of reactive oxygen species in the bone microenvironment [20]. Ageing and decrease in ovarian function with menopause, the main causes of osteoporosis, have been associated with spontaneous increases in proinflammatory cytokines, resulting in chronic low-grade systemic inflammation [21, 22]. Ageing, menopause, and proinflammatory cytokines have been shown to regulate ROS levels in cells and tissues, leading to oxidative stress [23, 24]. On the other hand, oxidative stress may enhance the expression of genes involved in inflammation and lead to ageing and estrogen deficiency [25, 26]. These factors act synergistically to promote pathophysiological risks throughout the body and contribute to the onset of disease [27–29].

MSCs are adult stem cells with the potential to differentiate into mesodermal lineages and play an important role in tissue homeostasis and regeneration. During fracture healing, MSCs recruited from bone marrow or other adjacent tissues to the injured site undergo proliferation and differentiation into osteoblasts and chondrocytes and dominate the process of bone formation through intramembrane ossification and endochondral ossification [30]. During the early stages after fracture, the microenvironment at the fracture site experiences mild ischemia, hypoxia, and local inflammation due to blood vessel and soft tissue damage, leading to ROS accumulation and increased oxidative stress levels [31, 32]. Generally, these ROS can be cleared by the body’s antioxidant system, protecting surrounding cells, including MSCs, from oxidative damage [33]. Moreover, ROS at the physiological level may play important physiological roles in the process of fracture healing by targeting resident cells, including MSCs [34, 35]; however, during osteoporosis, when ROS levels exceed physiological levels, a high oxidative environment facilitates MSC senescence by inducing mitochondrial dysfunction, resulting in decreased proliferation, increased apoptosis, and decreased osteogenic differentiation [36–38], which may inhibit or delay the fracture healing process.Therefore, protecting MSCs from excessive oxidative impairment and promoting their differentiation ability during osteoporotic fracture healing are important.

Tanshinone IIA is a commonly used Chinese medicine that has significant effects on treating cardiovascular system diseases. Previous studies have revealed the beneficial effects of Tanshinone IIA in both healthy animals and animal models of bone loss [39–44]. However, there are few studies on the use of Tanshinone IIA for bone repair. In addition, most in vitro studies have focused on the function of Tanshinone IIA in inhibiting osteoclastogenesis [39–41], with little emphasis on its role in MSC osteogenic differentiation or osteoblast survival. Tanshinone IIA (1µM and 5µM) promoted the osteogenesis of mouse bone marrow MSCs at both the early and late stages; however, 20µM Tanshinone IIA inhibited osteogenesis [17]. In addition, it has been shown that Tanshinone IIA can reverse dexamethasone-induced cell apoptosis in MC3T3-E1 or H_2_O_2_-induced osteoblast apoptosis, which might be related to the inhibition of Nox4-mediated ROS production or the nuclear factor kappa-B signalling pathway [45, 46].

In this study, we used H_2_O_2_ to induce excessive oxidative stress in MSCs and confirmed that Tanshinone IIA at relatively low concentrations exerted protective effects by reversing H_2_O_2_-induced cell apoptosis and rescued H_2_O_2_-induced decreases in the osteogenic differentiation and mineralization of MSCs. Furthermore, we confirmed that Tanshinone IIA could increase antioxidant enzyme production and reduce ROS level in H_2_O_2_ treated MSCs, contributing to the antioxidative and antiapoptotic functions of Tanshinone IIA.

Keap1-Nrf2 signalling regulates the activation of various genes that protect cells from oxidative stress. Nrf2 activity is mainly regulated by Keap1 in response to oxidative stress. Under nonstress conditions, Nrf2 is sequestered in the cytoplasm by Keap1. However, under stress conditions, ROS interrupts Keap1-Nrf2 binding, which is followed by the nuclear translocation of Nrf2 and the transcription of target genes, including antioxidant enzymes [47, 48]. In the present study, we further clarified that Tanshinone IIA increased Nrf2 expression in MSCs and enhanced the production of antioxidant enzymes, including HO-1, CAT, and SOD1; however, the protein level of Keap1 was not significantly changed, suggesting that the regulation of Nrf2 by Tanshinone IIA may be independent of Keap1, highlighting the complexity of the underlying mechanisms involved.

It is now understood that Tanshinone IIA has limited bioavailability when administered orally [49], suggesting poor absorption or significant metabolism. Hydrogels are a group of materials with three-dimensional cross-linked network structures that can mimic the natural extracellular matrix, facilitating cell growth and nutrient transport. Chitosan/dextran-based hydrogels have been proven to have appropriate biocompatibility, biodegradability and mechanical properties, as well as negligible cytotoxicity, minimal swelling and definite efficacy as drug delivery vehicles [50–54]. In addition, it would be of great simplicity and clinical importance to treat patients with undisplaced fractures or repositioned fractures with an injection to the site of injury [55]. Therefore, we employed an injectable biodegradable hydrogel as a delivery system for the local release of Tanshinone IIA at fracture sites. By utilizing this approach in a fracture model using OVX mice, we demonstrated that the local application of Tanshinone IIA promoted fracture healing and improved the biomechanical properties of the bones. Furthermore, this treatment approach could be applied to patients with osteoporotic fractures.

In conclusion, we provided compelling evidence that Tanshinone IIA could promote Nrf2 expression and activate antioxidant enzymes during H_2_O_2_-induced oxidative stress to protect MSCs from cell apoptosis and osteogenic differentiation inhibition. In vivo, local application of Tanshinone IIA accelerated fracture healing in OVX mice.

Although antioxidation appears to be one of the mechanisms through which Tanshinone IIA facilitates this process, it is highly conceivable that multiple other pathways are likely involved. These effects may include anti-inflammatory effects and the promotion of angiogenesis. Further investigations are required to fully elucidate the comprehensive mechanisms underlying the regulatory effects of Tanshinone IIA on fracture healing.

## Data Availability

Dataset available on request from the authors.

## Abbreviations

MSC: Mesenchymal stem cells
OVX: Ovariectomized
ROS: Reactive oxygen species
Keap1: Kelch-like ech-associated protein-1
Nrf2: Nuclear factor erythroid 2-related factor 2
DMSO: Dimethyl sulfoxide
MEM: Minimum essential medium
RT-PCR: Reverse transcriptase polymerase chain reaction
SOD: Superoxide dismutase
HO-1: Heme oxygenase-1
CAT: Catalase
H_2_DCFDA: 2,7-dichlorodihydrofluorescein diacetate
DAPI: 4,6-diamidino-2-phenylindole
PBS: Phosphate buffered saline
CT: Computed tomography
BV: Bone volume
TV: Total volume
BMD: Bone mineral density
Tb. N: Trabecular number
Tb. Th: Trabecular thickness
Tb. Sp: Trabecular separation
TUNEL: TdT-mediated dUTP nick end labeling
ANOVA: Analysis of variance
ALP: Alkaline phosphatase
Runx2: Runt-related transcription factor 2
